# Dynamical time-reversal symmetry breaking and photo-induced chiral spin liquids in frustrated Mott insulators

**DOI:** 10.1038/s41467-017-00876-y

**Published:** 2017-10-30

**Authors:** Martin Claassen, Hong-Chen Jiang, Brian Moritz, Thomas P. Devereaux

**Affiliations:** 1 Department of Applied Physics, Stanford University, Stanford, CA 94305 USA; 2 Stanford Institute for Materials and Energy Sciences, SLAC & Stanford University, Stanford, CA 94025 USA; 3 Geballe Laboratory for Advanced Materials, Stanford University, Stanford, CA 94305 USA

## Abstract

The search for quantum spin liquids in frustrated quantum magnets recently has enjoyed a surge of interest, with various candidate materials under intense scrutiny. However, an experimental confirmation of a gapped topological spin liquid remains an open question. Here, we show that circularly polarized light can provide a knob to drive frustrated Mott insulators into a chiral spin liquid, realizing an elusive quantum spin liquid with topological order. We find that the dynamics of a driven Kagome Mott insulator is well-captured by an effective Floquet spin model, with heating strongly suppressed, inducing a scalar spin chirality **S**_*i*_ · (**S**_*j*_ × **S**_*k*_) term which dynamically breaks time-reversal while preserving SU(2) spin symmetry. We fingerprint the transient phase diagram and find a stable photo-induced chiral spin liquid near the equilibrium state. The results presented suggest employing dynamical symmetry breaking to engineer quantum spin liquids and access elusive phase transitions that are not readily accessible in equilibrium.

## Abstract (web summary)

Exotic quantum phases like spin liquids have long been investigated theoretically but it is difficult to find materials that realize these states in equilibrium. Here the authors propose that optical driving could be used to induce chiral spin liquid behaviour in frustrated Mott insulators.

## Introduction

Control of quantum materials out of equilibrium represents one of the grand challenges of modern condensed matter physics. While an understanding of general non-equilibrium settings beyond heating and thermalization is still in its infancy, a loophole concerns considering instead the transient quantum states of quasi-periodic perturbations such as wide-envelope laser pulses. Here, much of the intuition and language of equilibrium survives in a distinctly non-equilibrium setting within the framework of Floquet theory. While recently enjoying much attention and experimental success in the manipulation of single-particle spectra^1–4^ and band topology or short-range entangled topological states^5–7^, a natural extension regards pumping of strongly correlated systems. Here, the essence of Floquet physics lies not merely in imbuing one-^1^ and two-particle^2^ responses with the pump frequency as an additional energy scale, but in reshaping the underlying Hamiltonian to stabilize phases of matter that might be inaccessible in equilibrium.

Indeed, initial investigations suggest that the notion of effective low-energy physics persists in certain high-frequency regimes of time-periodic perturbations, leading for instance to enhancement of correlated hopping^8, 9^, strong-field sign reversal of nearest-neighbor Heisenberg exchange in a 1D magnet^10, 11^, or enhancement of Cooper-pair formation^12–14^. Similar ideas are being pursued in the field of ultracold atoms to simulate artificial gauge fields, to dynamically realize topological band structures^15^ or even propose fractional quantum Hall effects and spin liquids in optical lattices^16, 17, 18^. At the same time, recent advances in Floquet thermodynamics indicate that, while driven non-integrable closed systems are in principle expected to heat up to infinite temperature^19, 20^, heating can be exponentially slow on pre-thermalized time scales^21–26^ or altogether avoided via many-body localization^27–29^ or dissipation^30–32^. An ideal condensed-matter realization hence entails a charge gap to limit absorption, as well as a delicate balance of competing phases, such that time-dependent perturbations and dynamical symmetry breaking can be expected to have an outsized effect and phase boundaries can be reached on pre-thermalized time scales with moderate effort.

Frustrated quantum magnets^33^ are prime candidates for such ideas. Strong local Coulomb repulsion between electrons freezes out the charge degrees of freedom, whereas the spin degrees of freedom are geometrically obstructed from ordering, hosting a delicate competition of conventionally ordered phases as well as quantum spin liquids (QSLs) with long-range entanglement and exotic excitations^34–41^. The chiral spin liquid (CSL) constitutes one of the earliest proposals of a topologically ordered QSL; it breaks time-reversal symmetry (TRS) and parity, while preserving SU(2) spin symmetry, and can be regarded as a bosonic *ν* = 1/2 fractional quantum Hall state of spins with zero net magnetization and gapped semion excitations^42–45^. While an unlikely ground state in unperturbed microscopic models, recently the CSL was found to be a competing state^46–53^, in particular after explicit breaking of TRS and parity^46–50^. However, TRS breaking in experiment is realized canonically via external magnetic fields, necessarily entailing a Zeeman shift as the dominant contribution, which breaks SU(2) symmetry and disfavors CSLs^46^.

Here, we show that pumping a Mott insulator with circularly polarized light below the Mott gap can dynamically break TRS without breaking of SU(2) or translation symmetry, providing a knob to drive a frustrated quantum magnet into a CSL. Starting from a prototypical Hubbard model, the key questions posed by this work are three-fold: First, how does optically induced TRS breaking manifest itself in a Mott insulator; second, can the ensuing effective Floquet spin model support a transient CSL and what are its signatures; and finally, does such an effective Floquet steady-state description capture the many-body time evolution of an optically driven Hubbard model? In the following, we answer all three questions affirmatively.

## Results

### Floquet-Hubbard model

Our focus lies on Kagome antiferromagnets, which have recently garnered much attention due to candidate materials herbertsmithite, kapellasite, and others^34^ with putative spin-liquid behavior at low temperatures. Experiments^54^ and first-principles calculations^55, 56^ indicate that the ground state and low-energy excitation spectra of these materials are well-captured by antiferromagnetic Heisenberg exchange between *d* ^9^ spins localized on Cu^34^. However, as photons couple to charge, a microscopic modeling of the light-matter interaction in principle must account for the multi-orbital structure at higher energies^57^, above the ~2 eV charge gap^58^. Here, we take a phenomenological approach, and, as an effective starting point that captures the essential physics but without pretense of a direct materials connection, start from a driven single-orbital Hubbard model at half filling

1 $$\hat H(t) = - {t_{\rm{h}}}\mathop {\sum}\limits_{\left\langle {ij} \right\rangle \sigma } {e^{i\frac{e}{\hbar }\,{{\bf{r}}_{ij}} \cdot {\bf{A}}(t)}} \hat c_{i\sigma }^\dag {\hat c_{j\sigma }} + U\mathop {\sum}\limits_i {\hat n_{i \uparrow }}{\hat n_{i \downarrow }}$$

Here, *t*_h_, *U*, *e* denote nearest-neighbor hopping, Coulomb interaction, and electron charge, **r**_*ij*_ denotes vectors between sites *i*,*j*, and **A**(*t*) = *A*(*t*)[cos(*Ωt*), sin(*Ωt*)]^*T*^ models a circularly polarized pump beam with wide-pulse envelope *A*(*t*), coupling to electrons via Peierls substitution. Comparison of nearest-neighbor exchange $J \approx 4t_{\rm{h}}^{\rm{2}}{\rm{/}}U$ with first-principles predictions for herbertsmithite^56^ suggests *U*/*t*_h_ of up to 40 due to the exceedingly narrow width of Cu *d*-orbital derived bands.

If *A*(*t*) varies slowly with respect to the pump period, then the Hamiltonian becomes approximately periodic under a translation $\hat H\left( {t + 2\pi {\rm{/}}\Omega } \right) = \hat H(t)$. Floquet theory then dictates that the behavior near the pump plateau is completely determined via many-body eigenstates of the form $\left| {{\Psi _n}(t)} \right\rangle = {e^{ - i{\epsilon _n}t}}\mathop {\sum}\nolimits_m {e^{im\Omega t}}\left| {{\Phi _m}} \right\rangle$ with ${\epsilon _n}$ the Floquet quasi-energy, where the $\left| {{\Phi _m}} \right\rangle$ conveniently follow as eigenstates of the static Floquet-Hubbard Hamiltonian

2 $$\begin{array}{*{20}{l}} {\hat H} = & { - {t_{\rm{h}}} \mathop {\sum}\limits_{{\scriptstyle \left\langle {ij} \right\rangle \sigma \atop \scriptstyle mm \prime}} {{\cal J}_{m - m \prime}}\left( A \right){e^{i\left( {m - m\prime} \right)\mathrm{arg}{{\bf{r}}_{ij}}}} \, \hat c_{i\sigma }^\dag {{\hat c}_{j\sigma }} \otimes \left| m \right\rangle \left\langle {m \prime} \right|} \\ \\ & { + U \mathop {\sum}\limits_i {{\hat n}_{i \uparrow }}{{\hat n}_{i \downarrow }} \otimes {\bf{1}} - \mathop {\sum} \limits_m m \Omega \,{\bf{1}} \otimes \left| m \right\rangle \left\langle m \right|} \end{array}$$

where *A* denotes the dimensionless field strength at the pump plateau, such that *A*(*t*) ≈ *Aħ*/(*ea*_0_) with *a*_0_ the nearest-neighbor distance, $m \in {\Bbb Z}$ is the Floquet index, and ${{\cal J}_m}( \cdot )$ denotes the Bessel function of the first kind (Methods section). Note that the apparent Hilbert space expansion is merely a gauge redundancy of Floquet theory, as eigenstates with energy ${\epsilon _n}$ + *mΩ* identify with the same physical state ∀*m*.

### Floquet Chiral spin model

Physically, Eq. (2) describes photon-assisted hopping in the presence of interactions, where electrons can enlist *m* photons to hop at a reduced energy cost *U* − *mΩ* of doubly occupying a site. Deep in the Mott phase the formation of local moments persists out of equilibrium as long as the pump remains off resonance and red-detuned from the charge gap. However, photon-assisted hopping reduces the energy cost of virtual exchange, pushing the system closer to the Mott transition and enlarging the range of virtual hopping paths that provide non-negligible contributions to longer-ranged exchange or multi-spin processes. Second, electrons acquire gauge-invariant phases when hopping around loops on the lattice for circular polarization. Crucially, and in contrast to an external magnetic field, an optical pump precludes a Zeeman shift, retaining the *SU*(2) spin rotation symmetry that is shared by CSL ground states. Symmetry considerations dictate that a manifestation of TRS breaking must to lowest-order necessarily involve a photo-induced scalar spin chirality *χ*_*ijk*_ term, with:

3 $${\hat H_{{\rm{spin}}}} = \mathop {\sum}\limits_{ij} {J_{ij}} {{\bf{S}}_i} \cdot {{\bf{S}}_j} + \mathop {\sum}\limits_{ijk} {\chi _{ijk}} {{\bf{S}}_i} \cdot \left( {{{\bf{S}}_j} \times {{\bf{S}}_k}} \right)$$

**Fig. 1:**
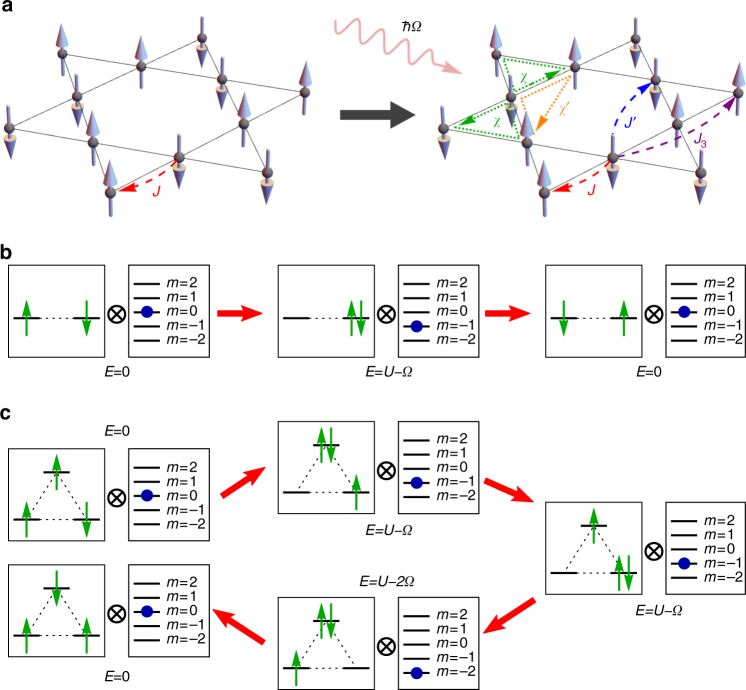
Photo-induced Kagome chiral antiferromagnet. **a** Starting from a Kagome Mott antiferromagnet, pumping with circularly polarized light dynamically breaks time-reversal and parity while preserving SU(2) spin symmetry, photo-inducing scalar spin chirality **S**_*i*_ · (**S**_*j*_ × **S**_*k*_) contributions on elementary equilateral (*χ*) and isosceles (*χ*′) triangles. Pump strength and frequency provide knobs to tune *χ*,*χ*′ as well as Heisenberg exchange *J*, *J*′, *J*_3_, as described in the main text. Examples of **b** nearest-neighbor and **c** three-site Floquet virtual hopping processes including absorption of photons, in the Mott-insulating regime. Boxes graphically depict the example initial, virtual intermediate and final states for second- and fourth-order virtual hopping processes, in terms of the product space of electronic degrees of freedom and the Floquet index. TRS is broken in **c**, inducing scalar spin chiralities *χ* on triangles of the lattice, whereas **b** solely induces nearest-neighbor Heisenberg exchange

This Floquet Chiral Spin Hamiltonian is the central focus of the paper; to derive it microscopically from the driven Kagome-Hubbard model (1), it is instructive to first consider the high-frequency limit $\Omega \gg U,{t_{\rm{h}}}$. Here, circularly polarized pumping induces complex nearest-neighbor hoppings $\tilde t = {t_{\rm{h}}}\left( {1 - {A^2}{\rm{/}}4} \right) + i\left( {\sqrt 3 {\rm{/}}4} \right)t_{\rm{h}}^{\rm{2}}{A^2}{\rm{/}}\Omega$ as well as purely-complex next-nearest-neighbor hoppings $\tilde t' = - i\left( {\sqrt 3 {\rm{/}}4} \right)t_{\rm{h}}^2{A^2}{\rm{/}}\Omega$, analogous to a staggered magnetic flux pattern in the unit cell (Supplementary Note 1). To third order in $\tilde t,\tilde t'$, a spin description then includes scalar spin chirality contributions, with $\chi = 9\sqrt 3 t_{\rm{h}}^4{A^2}{\rm{/}}2{U^2}\Omega$ of equal handedness for both equilateral triangles per unit cell, as depicted in Fig. 1a, and six isosceles triangles of opposite handedness with *χ*′ = *χ*/3, such that the total chiral couplings in the unit cell sum to zero.

Now consider sub-gap pumping *Ω* < *U*. Starting from Eq. (2), a microscopic derivation of the Floquet spin Hamiltonian proceeds via quasi-degenerate perturbation theory, where care must be taken to simultaneously integrate out *m *≠ 0 Floquet states and many-body states with doubly occupied sites (Supplementary Note 2). Figure 1b, c depict relevant virtual processes, involving simultaneous hopping of electrons and absorption of *m* photons with intermediate energy cost *U* − *mΩ*. To second order in virtual hopping, two-site exchange processes (Fig. 1b) are phase-agnostic and solely renormalize the nearest-neighbor Heisenberg exchange $\tilde J = 4\mathop {\sum}\nolimits_m {\left| {{{\cal J}_m}\left( A \right)} \right|^2}t_{\rm{h}}^2{\rm{/}}\left( {U - m\Omega } \right)$ ^8, 10^. While every process contributes to Heisenberg exchange, a scalar spin chirality contribution appears for multi-hop processes that enclose an area. Naïvely, to third order, an electron could simply circumnavigate the elementary triangles of the Kagome lattice; however, these processes interfere destructively and cancel exactly to all orders in *A* even though TRS is broken, and in contrast to an external magnetic field (Supplementary Note 2). This is consistent with results on the resonant A_2*g*_ Raman response of Mott insulators^59^, that connect to the *A* → 0, *m* = 1 limit. Instead, TRS breaking first manifests itself to fourth order in virtual hopping. Here, processes (Fig. 1c) can either encompass an elementary triangle, or virtually move an electron back and forth two legs of a hexagon, inducing scalar spin chirality contributions as shown in Fig. 1a, with

4 $$\chi = 3\mathop {\sum}\limits_{\bf{m}} \left[ {{\rm{sin}}\left[ {{\textstyle{{2\pi \left( {{m_1} - {m_2} + {m_3}} \right)} \over 3}}} \right]\Lambda _{\bf{m}}^{(1)} - {\rm{sin}}\left[ {{\textstyle{{2\pi {m_2}} \over 3}}} \right]\Lambda _{\bf{m}}^{(2)}} \right]$$

5 $$\chi ' = \mathop {\sum}\limits_{\bf{m}} \left[ {{\rm{sin}}\left[ {{\textstyle{{\pi \left( {{m_1} - {m_2} + {m_3}} \right)} \over 3}}} \right]\Lambda _{\bf{m}}^{(1)} - {\rm{sin}}\left[ {{\textstyle{{\pi {m_2}} \over 3}}} \right]\Lambda _{\bf{m}}^{(2)}} \right]$$

where **m** = {*m*_1_, *m*_2_, *m*_3_} are Floquet indices, and

6 $$\Lambda _{\bf{m}}^{(1)} = \frac{{8t_{\rm{h}}^4{{\cal J}_{{m_1}}}\left( A \right){{\cal J}_{{m_2} - {m_1}}}\left( A \right){{\cal J}_{{m_2} - {m_3}}}\left( A \right){{\cal J}_{{m_3}}}\left( A \right)}}{{\mathop {\prod}\nolimits_{i = 1}^3 \left( {U - {m_i}\Omega } \right)}}$$

7 $$\Lambda _{\bf{m}}^{(2)} = 2\left( {1 - {\delta _{{m_2}}}} \right){\textstyle{{U - {m_2}\Omega } \over {{m_2}\Omega }}}{\left( { - 1} \right)^{{m_1} - {m_3}}}{\rm{co}}{{\rm{s}}^2}\left[ {{\textstyle{{{m_2}\pi } \over 2}}} \right]\Lambda _{\bf{m}}^{(1)}$$

Here, $\Lambda _{\bf{m}}^{(1)}$ and $\Lambda _{\bf{m}}^{(2)}$ parameterize fourth-order virtual hopping processes for which the second intermediate virtual state retains a single double-occupied site or returns to local half filling (albeit with non-zero Floquet index), respectively. Furthermore, next-nearest-neighbor Heisenberg exchange

8 $$\begin{array}{l} J' = \mathop {\sum}\limits_{\bf{m}} \left[ {{\Gamma _{\bf{m}}} - {\rm{cos}}\left[ {{\textstyle{{\pi \left( {{m_1} - {m_2} + {m_3}} \right)} \over 3}}} \right]\frac{{\Lambda _{\bf{m}}^{(1)}}}{2} + {\rm{cos}}\left[ {{\textstyle{{\pi {m_2}} \over 3}}} \right]\frac{{\Lambda _{\bf{m}}^{(2)}}}{2}} \right]\\ {J_3} = \mathop {\sum}\limits_{\bf{m}} \left[ {{\Gamma _{\bf{m}}} - \frac{{\Lambda _{\bf{m}}^{(1)}}}{2} + \frac{{\Lambda _{\bf{m}}^{(2)}}}{2}} \right]\\ \end{array}$$

and corrections to nearest-neighbor Heisenberg exchange

9 $$\begin{array}{*{20}{l}} J \hfill & = \hfill & {\tilde J + \mathop {\sum}\limits_{\bf{m}} \left\{ {\left[ {2 + {\rm{cos}}\left[ {{\textstyle{{\pi \left( {{m_1} - {m_3}} \right)} \over 3}}} \right] + {\rm{cos}}\left[ {{\textstyle{{2\pi \left( {{m_1} - {m_3}} \right)} \over 3}}} \right] } \right.} \right.} \hfill \\ {} \hfill & {} \hfill & {\left. { + {\rm{cos}}\left[ {{\textstyle{{\pi \left( {{m_1} - {m_2} + {m_3}} \right)} \over 3}}} \right] + {\textstyle{1 \over 2}} \, {\rm{cos}}\left[ {{\textstyle{{2\pi \left( {{m_1} - {m_2} + {m_3}} \right)} \over 3}}} \right]} \right]\Lambda _{\bf{m}}^{(1)}} \hfill \\ {} \hfill & {} \hfill & {\left. { - \left[ {1 + 2\,{\rm{cos}}\left[ {\pi {m_2}} \right] + {\rm{cos}}\left[ {{\textstyle{{\pi {m_2}} \over 3}}} \right] + {\textstyle{1 \over 2}}{\rm{cos}}\left[ {{\textstyle{{2\pi {m_2}} \over 3}}} \right]} \right]\Lambda _{\bf{m}}^{(2)} - 7{\Gamma _{\bf{m}}}} \right\}} \hfill \\ \end{array}$$

appear at the same order, with

10 $${\Gamma _{\bf{m}}} = 4t_{\rm{h}}^4\left[ {\frac{{{\delta _{{m_3},0}}}}{{U - {m_1}\Omega }} + \frac{{{\delta _{{m_3},0}}}}{{U - {m_2}\Omega }}} \right]\mathop {\prod}\limits_{i = 1}^2 \frac{{{{\left[ {{{\cal J}_{{m_i}}}\left( A \right)} \right]}^2}}}{{\left( {U - {m_i}\Omega } \right)}}$$

parameterizing a twofold virtual nearest-neighbor exchange process.

### Steady-state phase diagram

**Fig. 2:**
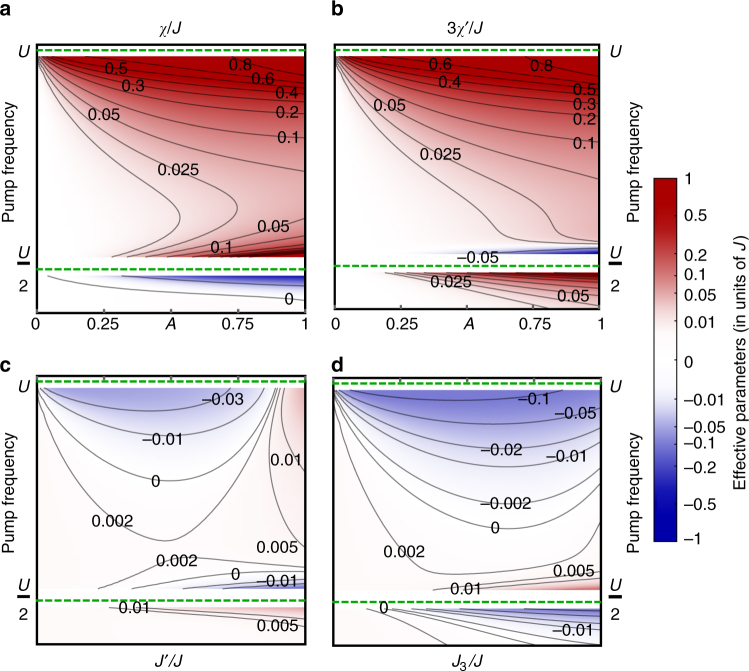
Pump dependence of chiral spin model parameters. Photo-induced time-reversal symmetry (TRS) breaking scalar spin chirality *χ*, *χ*′ (**a**, **b**) and TRS preserving Heisenberg exchange *J*′, *J*_3_ (**c**, **d**) terms for a Kagome Mott insulator pumped by circularly polarized light, as a function of pump strength *A* and pump frequency (from Eqs. (4)–(10)). Parameters are depicted in units of nearest-neighbor exchange coupling *J*. *Dashed lines* indicate one- and two-photon resonances with respect to the Hubbard repulsion *U*. Equal signs of *χ*, *χ*′ signify a staggering of **S**_*i*_ · (**S**_*j*_ × **S**_*k*_) contributions between elementary triangles and isosceles triangles inside the hexagons

Having established the effective steady-state physics for the duration of the pump pulse, the next question concerns whether the photo-induced Floquet spin model (Eq. (3)) can indeed stabilize a CSL. Consider its parameter space as a function of *A*, *Ω*, depicted in Fig. 2 for *U* = 20*t*_h_. Adiabatic ramping up of the circularly polarized pump then corresponds to horizontal trajectories with fixed *Ω*. Figure 2a, b show that TRS-breaking scalar spin chiralities develop with increasing field strength, whereas the effect on longer-ranged Heisenberg exchange ((c) and (d)) is comparatively weak. This immediately suggests that circularly polarized pumping grants a handle to change the underlying many-body state. Close to the one-photon resonance (*Ω* = *U*), chiral contributions are staggered between elementary and isosceles triangles (Fig. 1a), whereas *χ*′ changes sign when *Ω* approaches a two-photon resonance (*Ω* = *U*/2).

**Fig. 3:**
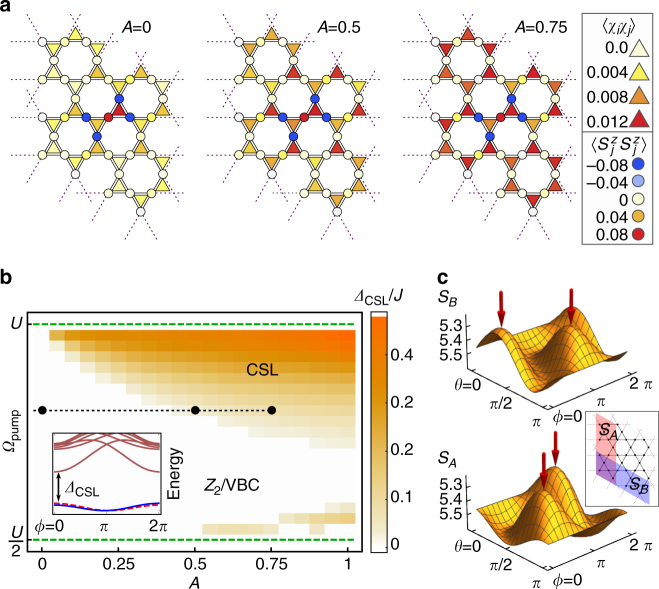
Identification of a photo-induced chiral spin liquid. **a** Evolution of spin $\left\langle {{{\bf{S}}_i} \cdot {{\bf{S}}_j}} \right\rangle$ (*circles* on vertices) and chiral $\left\langle {\left[ {{{\bf{S}}_i} \cdot \left( {{{\bf{S}}_j} \times {{\bf{S}}_k}} \right)} \right]\left[ {{{\bf{S}}_l} \cdot \left( {{{\bf{S}}_m} \times {{\bf{S}}_n}} \right)} \right]} \right\rangle$ (*triangles*) correlation functions as a function of pump strength *A*, from 36-site exact diagonalization of Eq. (3). **b** Minimum chiral spin liquid (CSL) excitation gap *Δ*_CSL_ above twofold degenerate ground states, under flux insertion through the torus. Inset depicts the twofold quasi-degenerate ground states (*blue* and *dashed-red lines*) and gap to excited states (*brown lines*), as a function of flux *ϕ* threaded through the torus (Methods section). The equilibrium ground state (a putative *Z*_2_ spin liquid or valence bond crystal) transitions into a photo-induced CSL at finite pump strength. **c** Rényi entropies and entanglement minima, of CSL ground state superpositions ${\rm{cos}}(\theta )\left| {{\psi _1}} \right\rangle + {\rm{sin}}(\theta ){e^{i\phi }}\left| {{\psi _2}} \right\rangle$ for two bipartitions of the 36-site torus (inset), discussed in the main text

We analyze the steady-state phase diagram using exact diagonalization of the Floquet spin model (Eq. (3)), parameterized by pump strength and frequency. In equilibrium (*A* = 0), the ground state of Eq. (3) is gapped and TRS invariant. Absence of conventional spin order is evidenced by a rapid decay of spin–spin and chiral–chiral correlation functions on a 36-site cluster (Fig. 3a), consistent with density-matrix renormalization group simulations that find a gapped *Z*_2_ QSL^60–63^. We adopt this view for the thermodynamic limit, but note that the ground state degeneracy of a *Z*_2_ QSL remains inaccessible in exact diagonalization of finite-size clusters (Methods section). Upon pumping (*A* ≠ 0), the spin correlator displays no propensity for ordering; however, chiral correlations develop smoothly (Fig. 3a). Importantly, a twofold ground state quasi-degeneracy develops continuously, with a gap to many-body excitations, indicative of a CSL. To track the phase boundary as a function of *A*, *Ω*, we determine the parameter space region within which the ground state degeneracy as well as the gap *Δ*_CSL_ to the many-body excitation manifold above the CSL survives insertion of a flux quantum through the torus (Methods section). As shown in Fig. 3b, a robust photo-induced CSL develops already for weak *A*, with excited states well-separated in energy. Finally, a proper verification of the CSL necessitates characterizing its ground state topological order. We therefore fingerprint the photo-induced phase by determining a basis of minimally entangled states from combinations of the two degenerate ground states $\left| {{\psi _{1,2}}} \right\rangle$, minimizing the Rényi entropy for their reduced density matrices in two distinct bipartitions (Fig. 3d) on a 36-site torus (Methods sections). *C*_6_ symmetry allows extraction of both modular ${\cal U}$, ${\cal S}$ matrices^64–66^ that encode self- and mutual-braiding statistics of the elementary excitations. We find that the photo-induced CSL corresponds uniquely to a $\nu = {\textstyle{1 \over 2}}$ bosonic fractional quantum Hall state, with matching modular matrices^40^

11 $${\cal U} = {\rm e^{ - \, i\frac{{2\pi }}{{24}} \times 1.02}}\left[ {\begin{array}{*{20}{c}} 1 & 0 \\ 0 & {0.99i} \\ \end{array}} \right],\,{\cal S} = {\textstyle{1 \over {\sqrt 2 }}}\left[ {\begin{array}{*{20}{c}} {1.00} & {1.00} \\ {1.00} & { - 1.02} \\ \end{array}} \right]$$

### Time evolution

Having established a photo-induced CSL for the Floquet spin model, the final question concerns whether this effective spin model qualitatively captures the time evolution of the driven Hubbard model (Eq. (1)).

**Fig. 4:**
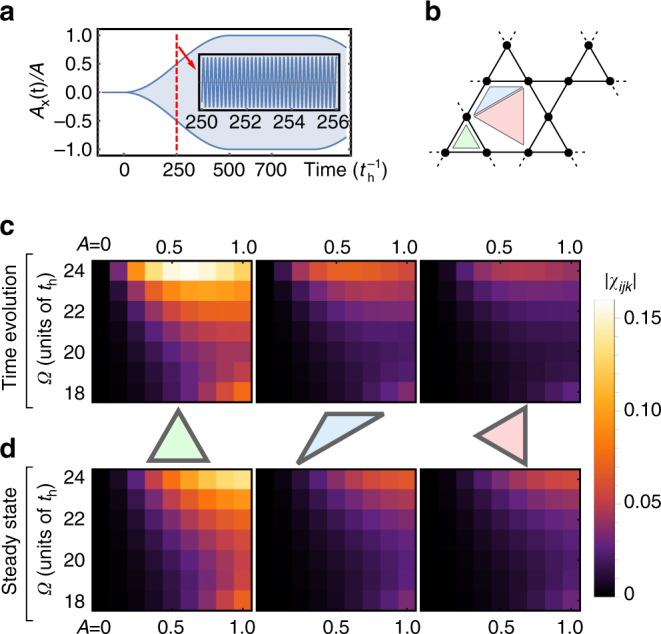
Time evolution for broad-pulse irradiation. Comparison of the Floquet chiral spin model (Eq. (3)) and the exact time evolution of the 12-site *U* = 30 Kagome Hubbard model (Eq. (1)) driven by circularly polarized light with ultra-slow adiabatic pump envelopes **a**. The scalar spin chiralities ${\chi _{ijk}} = \left\langle {{{\bf{S}}_i} \cdot \left( {{{\bf{S}}_j} \times {{\bf{S}}_k}} \right)} \right\rangle$ on elementary triangles of the unit cell **b** are depicted in **c**, **d**. **c** depicts *χ*_*ijk*_(*t*) measured from exact time evolution and averaged over the pump plateau; **d** depicts corresponding static expectation values of the Floquet spin model with Heisenberg and chiral couplings chosen as a function of *A*, *Ω*

To this end, we consider a circularly polarized optical pump pulse with a slow sinusoidal ramp-up and a wide pump plateau (Fig. 4a), and simulate the exact many-body dynamics of driven 12-site *U* = 30 Kagome Hubbard clusters for long times $t \le 1000\,t_{\rm{h}}^{ - 1}$. Conceptually, the transient state can then be thought of as dynamically following the instantaneous Floquet eigenstate $\left| {\Psi \left( {\tau ,{{\bar T}_{{\rm{slow}}}}} \right)} \right\rangle \approx {e^{ - i\epsilon \left( {{{\bar T}_{{\rm{slow}}}}} \right)\tau }}\mathop {\sum}\nolimits_m {e^{im\Omega \tau }}\left| {\Phi \left( {{{\bar T}_{{\rm{slow}}}}} \right)} \right\rangle$, with the time variable *t* “separating” into fast (*τ*) and slow $\left( {{{\bar T}_{{\rm{slow}}}}} \right)$ moving components. Reaching the pump plateau, the time-evolved state will nevertheless retain a finite quasi-energy spread $\left| {\Psi (t)} \right\rangle = \mathop {\sum}\nolimits_\alpha \rho_\alpha {e^{ - i{\epsilon _\alpha }t}}\mathop {\sum}\nolimits_m {e^{im\Omega t}}\left| {{\Phi _\alpha }(t)} \right\rangle$ (with *α* indexing the Floquet eigenstates). While dephasing of these constituent Floquet eigenstates should ultimately thermalize the system to infinite temperature, the system nevertheless matches the effective spin dynamics described by Eq. (3) and barely absorbs energy on the broad “pre-thermalized” time scales of interest, as we show below.

First, to compare to the Floquet spin description, we focus on time-dependent scalar spin chirality expectation values ${\chi _{ijk}}(t) = \left\langle {{{\bf{S}}_i} \cdot \left( {{{\bf{S}}_j} \times {{\bf{S}}_k}} \right)} \right\rangle$ $\left( {{{\bf{S}}_i} = \hat c_{i\sigma }^\dag {{\overrightarrow {\bf{s}} }_{\sigma \sigma '}}{{\hat c}_{i\sigma '}}} \right)$ on elementary triangles of the Kagome cluster (Fig. 4b). Vanishing in equilibrium due to TRS, the pump-period average of *χ*_*ijk*_(*t*) should saturate to its Floquet expectation value at the pump plateau. Figure 4c compares *χ*_*ijk*_(*t*), time-averaged over the pump plateau, to corresponding static *χ*_*ijk*_ expectation values of the Floquet spin model (3) ground state. The latter follows from choosing *χ*, *χ*′, *J*, *J*′, *J*_3_ via Eqs. (4)–(10), with *A*, *Ω* the pump parameters of the Hubbard time evolution. Intriguingly, the electronic time evolution is in excellent qualitative agreement with predictions for the Floquet spin model, even when driven close to the Mott transition.

Quantitative discrepancies predominantly originate from deviations of the local moment $\left\langle {{{\left( {{S^z}} \right)}^2}} \right\rangle < 1{\rm{/}}4$; additionally, the 12-site cluster with periodic boundary conditions permits weak ring exchange contributions from loops of virtual hopping around the cluster. Importantly, the transient increase in double occupancies is not an indication of heating—instead, this follows from a reduction of the effective *U* of the transient Floquet-Hubbard Hamiltonian, a consequence of the photo-assisted hopping processes depicted in Fig. 1b, c. Quantitative differences between spin and fermionic observables are therefore analogous to differences between canonical spin and fermionic descriptions of equilibrium quantum magnets for a finite Hubbard-*U*.

**Fig. 5:**
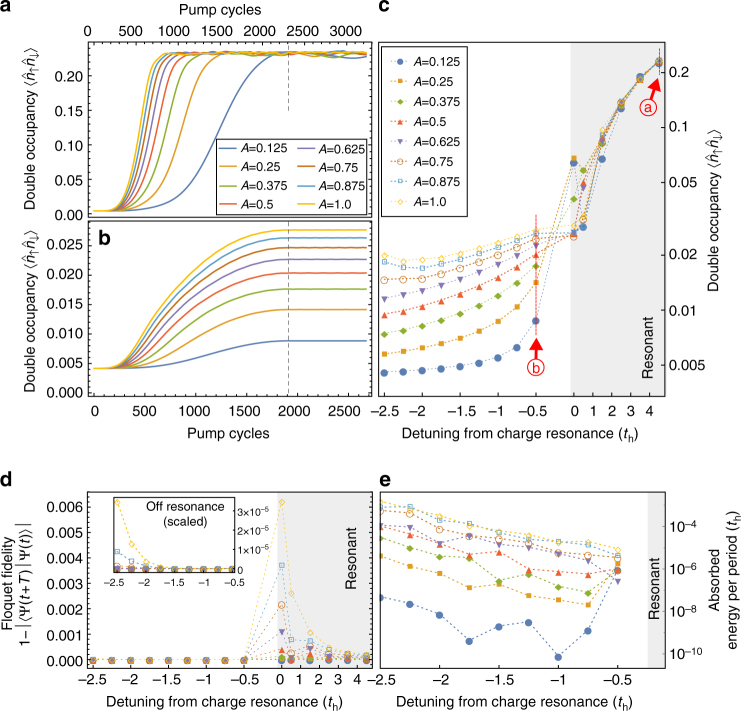
Pre-thermalization and transient Floquet steady states. **a**, **b** depict the time evolution of the period-averaged local double occupancy of the 12-site driven Hubbard model as a function of cycles under the pump, on and off the charge resonance with the upper Hubbard band, respectively. *Dashed lines* indicate onset of the pump plateau. **c** Extracted transient expectation values of the double occupancy at the pump plateau, as a function of pump strength *A* and detuning from the upper Hubbard band. *Red arrows* indicate values of *t*_h_ used for **a**, **b**. *Lines* are guides to the eye. On resonance (*gray* region), the system heats rapidly, with $\left\langle {{{\hat n}_ \uparrow }{{\hat n}_ \downarrow }} \right\rangle$ thermalizating independent of the pump strength *A* and approaching its infinite-temperature expectation value 1/4. Below resonance (*white region*), the system transiently realizes the TRS-breaking chiral quantum magnet, with a tunable Hubbard interaction *U* (as well as correspondingly tunable magnetic *J*, *χ*, see Fig. 4) as a function of pump strength. **d** Floquet fidelity $F(T) = \left| {\left\langle {\Psi \left( {t + T} \right)\left| {\Psi (t)} \right.} \right\rangle } \right|$ on the pump plateau—below charge resonance, (1 − *F*) → 0, indicating the controlled preparation of a Floquet eigenstate. **e** Extracted stroboscopic heating rates per pump cycle and below resonance. Remarkably, heating is strongly suppressed close to the charge resonance, with the driven system requiring many thousands of pump cycles to finally absorb energy on the order of the Heisenberg exchange *J*

To analyze this in detail, we focus on pumping the system across the charge resonance with the upper Hubbard band, where the photo-induced scalar spin chirality contribution is expected to be largest. Figure 5a–c show the period-averaged double occupancy $\left\langle {{{\hat n}_ \uparrow }{{\hat n}_ \downarrow }} \right\rangle$ as a function of pump strength and detuning from the charge resonance ≈*U* − 5.5*t*_h_. Upon resonant charge excitation, the system heats up rapidly and the double occupancy approaches its infinite-temperature limit $\left\langle {{{\hat n}_ \uparrow }{{\hat n}_ \downarrow }} \right\rangle \to 1{\rm{/}}4$. Importantly, this entails that thermalization at long times is independent of the pump strength *A*.

Conversely, in the off-resonant regime, one observes a pump-strength dependent saturation of the double occupancy. Here, proper heating is strongly suppressed and the system instead realizes the effective Floquet chiral quantum magnet with a transient reduction of the Hubbard interaction *U*. To verify that the driven steady state indeed follows the ground state of the effective Floquet Hamiltonian adiabatically, consider a period-shifted Floquet “fidelity measure” ${\cal F} = \left| {\left\langle {\Psi \left( {t + T} \right)\left| {\Psi (t)} \right.} \right\rangle } \right|$, where *T* = 2*π*/*Ω* is the pump period. At the pump plateau with discrete time-translation symmetry, ${\cal F}$ is time-independent and quantifies the Floquet quasi-energy spread of the transient steady state (Fig. 5d). For a pure Floquet state, $1 - {\cal F} \to 0$, suggesting that the driven state below resonance adiabatically follows a Floquet eigenstate, whereas adiabaticity is lost when crossing the absorption edge.

To distinguish residual heating on these pre-thermalized time scales of interest^25, 26^ from a transient increase in energy in the chiral quantum magnet due to modulation of the Hamiltonian, consider the period-averaged stroboscopic energy operator $\langle {\widehat E} \rangle$ (Methods section). On the pump plateau, both the double occupancy and $\langle {\widehat E} \rangle$ saturate to their pre-thermalized steady-state expectation values; however, a minuscule residual gradient over thousands of pump cycles remains. To good approximation for the time scales considered here, we can linearize the energy on the pump plateau $\langle {\widehat E} \rangle (t) \approx {E_0} + t\Delta E$, and extract the heating rate Δ*E* from simulations. Figure 5e depicts the absorbed energy per pump cycle on the pump plateau, as a function of pump strength and detuning from the absorption edge. Remarkably, residual heating is largely suppressed close to resonance, with an absorbed energy on the order of 10^−6^ *t*_h_ per pump cycle. Naïvely, this extraordinary meta-stability suggests that it could take on the order of tens of thousands of pump cycles for heating to dominate the dynamics, absorbing a total energy ~*J* the exchange coupling.

A more microscopic analysis of photo-excitation for realistic materials will likely lead to a less optimistic upper bound on the time scales of interest. First, a materials-specific modeling of electron–photon coupling and multi-band effects will modify the effective photo-induced spin Hamiltonian, albeit necessarily retaining the salient symmetry properties and scalar spin chirality contributions that stabilize the CSL. Second, an intriguing follow-up question regards the role of coupling to—and heating of—the lattice. While magneto-elastic coupling to phonons is weak in most materials and the optical frequencies under consideration are far from resonance with infrared-active phonon modes, electron phonon coupling will nevertheless indirectly heat the lattice due to Raman-assisted hopping processes. Conversely, the separation of time scales for electrons and phonons suggests that the phonon bath could similarly play the role of a dissipative channel, effectively “cooling” the electronic system. While initial investigations have already studied the case of free or weakly-interacting electrons^30–32^, a proper understanding of the confluence of strong interactions, external drive and dissipation remains an interesting topic for future study.

### Conclusions

In summary, we have shown that pumping a frustrated Mott insulator with circularly-polarized light can dynamically break TRS while preserving SU(2) symmetry of the underlying spin system, by augmenting its effective dynamics with a transient scalar spin chirality term. Remarkably, on the Kagome lattice this effective Floquet spin model was found to stabilize a transient CSL in a broad parameter regime. Our results suggest that wide-pulse optical perturbations can provide an intriguing knob to tune the low-energy physics of frustrated quantum magnets, shedding light on regions of their phase diagram hitherto unexplored.

## Methods

### Floquet theory

Consider a generic time-dependent many-body Hamiltonian with discrete time-translation invariance $\hat H(t) = \hat H\left( {t + 2\pi {\rm{/}}\Omega } \right)$. Instead of solving the many-body time evolution, one can reexpress the time-dependent Schrödinger equation in a Floquet eigenbasis $\left| {{\Phi _\alpha }(t)} \right\rangle = {e^{ - i{\epsilon _\alpha }t}}\mathop {\sum}\nolimits_m {e^{im\Omega t}}\left| {{u_{\alpha ,m}}} \right\rangle$, where *α* indexes the basis wave function and ${\epsilon _\alpha }$ is its respective Floquet quasi-energy. Then, determination of the time-dependent eigenstates of the driven system reduces to finding the time-independent eigenstates of the Floquet Hamiltonian

12 $${\hat H_{{\rm{floquet}}}} = \mathop {\sum}\limits_{mm'} \left[ {{{\hat H}_{m - m'}} - m\Omega {\delta _{m,m'}}} \right]\left| m \right\rangle \left\langle {m'} \right|$$

where ${\hat H_{m - m'}}$ are the Fourier expansion coefficients of $\hat H(t)$. Taking $\hat H(t)$ as the driven Kagome-Hubbard model of Eq. (1) in the main text straightforwardly recovers the Floquet-Hubbard Hamiltonian (Eq. (2)). Here, the dimensionless pump strength *A* that enters via Peierls substitution relates to the electric field ${\cal E}$ as ${\cal E} = \Omega {\rm{/}}\left( {e{a_0}} \right)$, with *e* the electron charge and *a*_0_ the nearest-neighbor bond distance. While realistic estimates for materials would necessarily entail significant contributions from multi-orbital effects and local dipole transitions, all of which are not captured within the single-orbital Hubbard model, a naïve estimate from solely Peierls substitution for a single-orbital approximation of herbertsmithite yields *A* = 0.25…0.5 for ${\cal E} \ldots 100 \ldots 200\,{\rm{meV}}\,{{\rm{{\AA}}}^{ - 1}}$ for a 900 nm near-infrared pump.

### Numerical simulations

To characterize the photo-induced CSL state, we performed exact diagonalization calculations of the Floquet chiral spin Hamiltonian, described in Eq. (3). Due to three-spin interactions and longer-ranged exchange interactions, the sparsity of the resulting Hamiltonian matrix is over an order of magnitude lower than for a nearest-neighbor Heisenberg antiferromagnet. Spin and chiral correlation functions as well as minimally entangled states were calculated for a 36-site cluster with periodic boundary conditions, spanned by vectors **R**_1_ = 4**a**_1_ − 2**a**_2_, **R**_2_ = −2**a**_1_ + 4**a**_2_, where **a**_1_, **a**_2_ are the lattice vectors. This choice retains the rotational symmetry of the Kagome lattice, facilitating extraction of the modular matrices. Previous exact diagonalization studies have simulated the 36-site cluster for a purely nearest-neighbor Heisenberg model^67, 68^. While density-matrix renormalization group studies indicate that such a model is likely to stabilize a *Z*_2_ QSL in the thermodynamic limit, it is well-known that its ground state degeneracy remains inaccessible in exact diagonalization of finite-size clusters^67, 68^, and the 36-site cluster is likely to host a QSL close to a phase boundary with a valence bond crystal^69^. However, the nature of the equilibrium ground state and its extrapolation to the thermodynamic limit does not affect the conclusions regarding transient state; crucially, the CSL state is well-stabilized already on the finite-size systems under consideration, with a robust many-body excitation gap.

The ground state degeneracy and minimal many-body excitation gap under flux insertion was calculated by imposing a spin-dependent twist of boundary conditions along one direction and tracking the many-body spectral flow as a function of twist angle, as depicted in the inset of Fig. 3b. A fine sampling of flux insertion, as depicted in Fig. 3b, was performed for 30-site clusters, spanned by vectors **R**_1_ = 2**a**_1_ + **a**_2_, **R**_2_ = −2**a**_1_ + 4**a**_2_, and checked against the 36-site cluster. The winding of the quasi-degenerate ground states upon flux insertion is a signature of CSLs, with the two quasi-degenerate ground states exchanging once under flux insertion, or remaining separated, depending on whether they lie in different (30-site cluster) or the same (36-site cluster) momentum sectors.

We furthermore consider two bipartitions *A*, *B* of the 36-site cluster with periodic boundary conditions, as depicted in the inset in Fig. 3c, and calculate the Rényi entropies

13 $${S_\alpha }(\theta ,\phi ) = - {\rm{log}}\,{\rm{tr}}\left\{ {\rho _\alpha ^2(\theta ,\phi )} \right\},\quad \alpha = A,B$$

where ${\rho _\alpha }(\theta ,\phi ) = {\rm{t}}{{\rm{r}}_\alpha }\left\{ {\left| {\Psi (\theta ,\phi )} \right\rangle \left\langle {\Psi (\theta ,\phi )} \right|} \right\}$ is the reduced density matrix on bipartition *α* = *A*, *B* for superpositions $\left| {\Psi (\theta ,\phi )} \right\rangle = {\rm{cos}}(\theta )\left| {{\psi _1}} \right\rangle + {\rm{sin}}(\theta ){e^{i\phi }}\left| {{\psi _2}} \right\rangle$ of the quasi-degenerate ground states $\left| {{\psi _1}} \right\rangle ,\left| {{\psi _2}} \right\rangle$, as a function of *θ*, *ϕ*. Figure 3c depicts *S*_*A*_ and *S*_*B*_ calculated from twofold quasi-degenerate ground states of the Floquet chiral spin model. For a CSL, *S*_*α*_(*θ*, *ϕ*) is expected to display two entanglement minima; the two corresponding minimally entangled states $\left| {\Psi (\theta ,\phi )} \right\rangle$ permit extraction of the modular matrices^64^, which match expectations for a Kalmeyer-Laughlin CSL and are quoted in the main text.

### Time evolution

The electronic many-body time evolution was simulated for a 12-site Kagome-Hubbard cluster (4 unit cells) with periodic boundary conditions, spanned by vectors **R**_1_ = 2**a**_1_, **R**_2_ = 2**a**_2_, and with the time propagation employing adaptive step size control. We note this is the minimum cluster size to faithfully host all permutations of virtual hopping processes that give rise to the effective Floquet chiral spin Hamiltonian discussed in the main text (Eq. (3)).

To model broad circularly polarized pump pulses, we consider a pulsed field

14 $${\bf{A}}(t) = A(t){\left[ {{\rm{cos}}(\Omega t),\,{\rm{sin}}(\Omega t)} \right]^{\rm T}}$$

with a smooth sinusoidal pump envelope

15 $$A(t) = \left\{ {\begin{array}{*{20}{l}} {0, } \hfill & {t \le 0} \hfill \\ {A\,{\rm{si}}{{\rm{n}}^2}\left( {\frac{\pi }{2}\frac{t}{{{t_{{\rm{plateau}}}}}}} \right), } \hfill & {0 < \, t < \, {t_{{\rm{plateau}}}}} \hfill \\ {A,} \hfill & {t \ge {t_{{\rm{plateau}}}}} \hfill \end{array}} \right.$$

where ${t_{{\rm{plateau}}}} = 700t_{\rm{h}}^{ - 1}$ for the results of the main text. Details on pump envelope dependence can be found in Supplementary Note 3.

Finally, to quantify energy absorption in the driven system, we compute the period-averaged energy operator

16 $$\begin{array}{*{20}{l}} {\hat E} \hfill & = \hfill & {\frac{1}{T}{\int}_t^{t + T} dt'\hat H\left( {t'} \right)} \hfill \\ {} \hfill & = \hfill & {U{{\hat n}_ \uparrow }{{\hat n}_ \downarrow } - {{\cal J}_0}(A){t_{\rm{h}}}\mathop {\sum}\limits_{\left\langle {ij} \right\rangle \sigma } \hat c_{i\sigma }^\dag {{\hat c}_{j\sigma }}} \hfill \end{array}$$

where ${{\cal J}_0}(A)$ denotes the zeroth Bessel function of the first kind. Note that $\langle {\widehat E} \rangle$ is time-independent for a pure Floquet state, in theory. Instead, the finiteness of the pump envelope entails a residual quasi-energy spread, with the resulting dephasing of the driven state leading to residual heating on the pump plateau. While the driven state is ultimately expected to thermalize to an infinite-temperature state at infinite times, the results of the main text demonstrate a long-lived and remarkably stable pre-thermalized regime with negligible absorption.

### Data availability

The data that support the results presented in this study are available from the corresponding authors on request.

## Electronic supplementary material

Supplementary Information

## References

[1] Wang YH, Steinberg H, Jarillo-Herrero P, Gedik N (2013). Observation of floquet-bloch states on the surface of a topological insulator. Science.

[2] Kim J (2014). Ultrafast generation of pseudo-magnetic field for valley excitons in Wse_2_ monolayers. Science.

[3] Sie EJ (2015). Valley-selective optical Stark effect in monolayer WS_2_. Nat. Mater..

[4] Mahmood F (2016). Selective scattering between FloquetâÂŞBloch and Volkov states in a topological insulator. Nat. Phys..

[5] Lindner NH, Refael G, Galitski V (2011). Floquet topological insulator in semiconductor quantum wells. Nat. Phys..

[6] Rudner MS, Lindner NH, Berg E, Levin M (2012). Anomalous edge states and the bulk-edge correspondence for periodically-driven two dimensional systems. Phys. Rev. X.

[7] Po HC, Fidkowski L, Morimoto T, Potter AC, Vishwanath A (2016). Chiral floquet phases of many-body localized bosons. Phys. Rev. X.

[8] Bukov M, Kolodrubetz M, Polkovnikov A (2016). Schrieffer-wolff transformation for periodically driven systems: strongly correlated systems with artificial gauge fields. Phys. Rev. Lett..

[9] Bukov M, Heyl M, Huse DA, Polkovnikov A (2016). Heating and many-body resonances in a periodically driven two-band system. Phys. Rev. B.

[10] Mentink JH, Balzer K, Eckstein M (2015). Ultrafast and reversible control of the exchange interaction in Mott insulators. Nat. Commun..

[11] Itin AP, Katsnelson MI (2015). Effective hamiltonians for rapidly driven many-body lattice systems: induced exchange interactions and density-dependent hoppings. Phys. Rev. Lett..

[12] Knap M, Babadi M, Refael G, Martin I, Demler E (2016). Dynamical Cooper pairing in non-equilibrium electron-phonon systems. Phys. Rev. B.

[13] Sentef MA, Kemper AF, Georges A, Kollath C (2016). Theory of light-enhanced phonon-mediated superconductivity. Phys. Rev. B.

[14] Coulthard J, Clark SR, Al-Assam S, Cavalleri A, Jaksch D (2017). Enhancement of superexchange pairing in the periodically driven Hubbard model. Phys. Rev. B.

[15] Jotzu G (2014). Experimental realization of the topological Haldane model with ultracold fermions. Nature.

[16] Yao NY (2012). Realizing fractional chern insulators with dipolar spins. Phys. Rev. Lett..

[17] Cooper NR, Dalibard J (2013). Reaching fractional quantum hall states with optical flux lattices. Phys. Rev. Lett..

[18] Yao, N. Y., Zaletel, M. P., Stamper-Kurn, D. M. & Vishwanath, A., A quantum dipolar spin liquid. Preprint at http://arxiv.org/abs/1510.06403 (2015).

[19] D’Alessio L, Rigol M (2014). Long-time behavior of isolated periodically driven interacting lattice systems. Phys. Rev. X.

[20] Lazarides A, Das A, Moessner R (2014). Equilibrium states of generic quantum systems subject to periodic driving. Phys. Rev. E.

[21] Canovi E, Kollar M, Eckstein M (2016). Stroboscopic prethermalization in weakly interacting periodically driven system. Phys. Rev. E.

[22] Kuwahara T, Mori T, Saito K (2016). Floquet-magnus theory and generic transient dynamics in periodically driven many-body quantum systems. Ann. Phys..

[23] Mori T, Kuwahara T, Saito K (2016). Rigorous bound on energy absorption and generic relaxation in periodically driven quantum systems. Phys. Rev. Lett..

[24] Bukov M, Gopalakrishnan S, Knap M, Demler E (2015). Prethermal floxquet steady states and instabilities in the periodically driven, weakly interacting bose-hubbard model. Phys. Rev. Lett..

[25] Abanin DA, De Roeck W, Huveneers F (2015). Exponentially slow heating in periodically driven many-body systems. Phys. Rev. Lett..

[26] Ho, W. W. & Abanin, D. A. Quasi-adiabatic dynamics and state preparation in Floquet many-body systems. Preprint at http://arxiv.org/abs/1611.05024 (2016).

[27] D’Alessio L, Polkovnikov A (2013). Many-body energy localization transition in periodically driven systems. Ann. Phys..

[28] Ponte P, Papić Z, Huveneers F, Abanin DA (2015). Many-body localization in periodically driven systems. Phys. Rev.Lett..

[29] Lazarides A, Das A, Moessner R (2015). Fate of many-body localization under periodic driving. Phys. Rev. Lett..

[30] Dehghani H, Oka T, Mitra A (2014). Dissipative Floquet topological systems. Phys. Rev. B.

[31] Iadecola T, Neupert T, Chamon C (2015). Occupation of topological Floquet bands in open systems. Phys. Rev. B.

[32] Seetharam KI, Bardyn C-E, Lindner N-H, Rudner M-S, Refael G (2015). Controlled population of Floquet-Bloch states via coupling to BOSE and Fermi Baths. Phys. Rev. X.

[33] Balents L (2010). Spin liquids in frustrated magnets. Nature.

[34] Norman MR (2016). Herbertsmithite and the search for the quantum spin liquid. Rev. Mod. Phys..

[35] Zhou Y, Kanoda K, Ng T-K (2017). Quantum spin liquid states. Rev. Mod. Phys..

[36] Yamashita M (2010). Highly mobile gapless excitations in a two-dimensional candidate quantum spin liquid. Science.

[37] Han T-H (2012). Fractionalized excitations in the spin-liquid state of a kagome-lattice antiferromagnet. Nature.

[38] Banerjee A (2016). Proximate Kitaev quantum spin liquid behaviour in a honeycomb magnet. Nat. Mater..

[39] Fu M, Imai T, Han T-H, Lee YS (2016). Evidence for a gapped spin-liquid ground state in a kagome Heisenberg antiferromagnet. Science.

[40] Wen X-G (1990). Topological orders in rigid states. Int. J. Mod. Phys. B.

[41] Wen X-G (2002). Quantum orders and symmetric spin liquids. Phys. Rev. B.

[42] Kalmeyer V, Laughlin RB (1987). Equivalence of the resonating-valence-bond and fractional quantum hall states. Phys. Rev. Lett..

[43] Kalmeyer V, Laughlin RB (1989). Theory of the spin liquid state of the Heisenberg antiferromagnet. Phys. Rev. B.

[44] Schroeter DF, Kapit E, Thomale R, Greiter M (2007). Spin Hamiltonian for which the chiral spin liquid is the exact ground state. Phys. Rev. Lett..

[45] Thomale R, Kapit E, Schroeter DF, Greiter M (2009). Parent Hamiltonian for the chiral spin liquid. Phys. Rev. B.

[46] Bauer B (2014). Chiral spin liquid and emergent anyons in a Kagome lattice Mott insulator. Nat. Commun..

[47] Wietek A, Sterdyniak A, Läuchli AM (2015). Nature of chiral spin liquids on the kagome lattice. Phys. Rev. B.

[48] Kumar K, Sun K, Fradkin E (2015). Chiral spin liquids on the kagome lattice. Phys. Rev. B.

[49] Hickey C, Cincio L, Papić Z, Paramekanti A (2016). Haldane-Hubbard mott insulator: from tetrahedral spin crystal to chiral spin liquid. Phys. Rev. Lett..

[50] Wietek A, Läuchli AM (2016). Chiral spin liquid and quantum criticality in extended *S* = 1/2 Heisenberg models on the triangular lattice. Phys. Rev. B.

[51] He Y-C, Sheng DN, Chen Y (2014). Chiral spin liquid in a frustrated anisotropic Kagome Heisenberg model. Phys. Rev. Lett..

[52] Gong S-S, Zhu W, Sheng DN (2014). Emergent chiral spin liquid: fractional quantum Hall effect in a Kagome Heisenberg model. Sci. Rep..

[53] Gong S-S, Zhu W, Balents L, Sheng DN (2015). Global phase diagram of competing ordered and quantum spin-liquid phases on the kagome lattice. Phys. Rev. B.

[54] Han T, Chu S, Lee YS (2012). Refining the spin Hamiltonian in the spin-1/2 Kagome lattice antiferromagnet ZnCu_3_(OH)_6_Cl_2_ using single crystals. Phys. Rev. Lett..

[55] Janson O, Richter J, Rosner H (2008). ModiïňĄed Kagome physics in the natural spin-1 = 2 Kagome lattice systems: kapellasite Cu_3_n(OH)_6_Cl_2_ and haydeeite Cu_3_Mg(OH)_6_Cl_2_. Phys. Rev. Lett..

[56] Jeschke HO, Salvat-Pujol F, Valentí R (2013). First-principles determination of Heisenberg Hamiltonian parameters for the spin-1/2 Kagome antiferromagnet ZnCu_3_(OH)_6_Cl_2_. Phys. Rev. B.

[57] Claassen M, Jia C, Moritz B, Devereaux TP (2016). All-optical materials design of chiral edge modes in transition-metal dichalcogenides. Nat. Commun..

[58] Helton JS (2007). Spin dynamics of the spin-1/2 kagome lattice antiferromagnet ZnCu_3_(OH)_6_Cl_2_. Phys. Rev. Lett..

[59] Ko W-H, Liu Z-X, Ng T-K, Lee PA (2010). Raman signature of the U(1) Dirac spin-liquid state in the spin-1/2 Kagome system. Phys. Rev. B.

[60] Jiang HC, Weng ZY, Sheng DN (2008). Density matrix renormalization group numerical study of the kagome antiferromagnet. Phys. Rev. Lett..

[61] Yan S, Huse DA, White SR (2011). Spin-liquid ground state of the *S* = 1/2 Kagome Heisenberg antiferromagnet. Science.

[62] Jiang HC, Wang Z, Balents L (2012). Identifying topological order by entanglement entropy. Nat. Phys..

[63] Depenbrock S, McCullough IP, Schollwöck U (2012). Nature of the spin-liquid ground state of the S = 1/2 Heisenberg model on the Kagome lattice. Phys. Rev. Lett..

[64] Zhang Y, Grover T, Turner A, Oshikawa M, Vishwanath A (2012). Quasiparticle statistics and braiding from ground-state entanglement. Phys. Rev. B.

[65] Cincio L, Vidal G (2013). Characterizing topological order by studying the ground states on an InïňĄnite cylinder. Phys. Rev. Lett..

[66] Zhu W, Sheng DN, Haldane FDM (2013). Minimal entangled states and modular matrix for fractional quantum Hall effect in topological flat bands. Phys. Rev. B.

[67] Läuchli AM, Sudan J, Sørensen ES (2011). Ground-state energy and spin gap of spin-1/2 Kagomé-Heisenberg antiferromagnetic clusters: large-scale exact diagonalization results. Phys. Rev. B.

[68] Nakano H, Sakai T (2011). Numerical-diagonalization study of spin gap issue of the Kagome lattice Heisenberg antiferromagnet. J. Phys. Soc. Jpn.

[69] Li, T. The spin-1/2 Heisenberg model on Kagome lattice as a quantum critical system. Preprint at http://arxiv.org/abs/1106.6134 (2011).

