# Supplementary material for: Dynamical time-reversal symmetry breaking and photo-induced chiral spin liquids in frustrated Mott insulators
Source: Nat Commun. 2017 Oct 30;8:1192. doi: 10.1038/s41467-017-00876-y (PMC5662750; doi:10.1038/s41467-017-00876-y)
Supplement: Supplementary file 1 — Supplementary Information [file 41467_2017_876_MOESM1_ESM.pdf]

Supplementary Note 1. PERTURBATION THEORY FOR THE NON-INTERACTING MODEL

Before discussing sub-gap pumping in the interacting Mott insulator, it is instructive to first consider electrons on the Kagome lattice in the absence of interactions, described by a Bloch Hamiltonian

$$h(\mathbf{k}) = -2t_{\mathrm{h}} \begin{bmatrix} 0 & \cos\left(\frac{\mathbf{a}_1\cdot\mathbf{k}}{2}\right) & \cos\left(\frac{\mathbf{a}_2\cdot\mathbf{k}}{2}\right) \\ \cos\left(\frac{\mathbf{a}_1\cdot\mathbf{k}}{2}\right) & 0 & \cos\left(\frac{\mathbf{a}_3\cdot\mathbf{k}}{2}\right) \\ \cos\left(\frac{\mathbf{a}_2\cdot\mathbf{k}}{2}\right) & \cos\left(\frac{\mathbf{a}_3\cdot\mathbf{k}}{2}\right) & 0 \end{bmatrix} \tag{1}$$

In equilibrium, the band structure mirrors graphene, with two Dirac points at the corners $\mathbf{K}, \mathbf{K}'$ of the Brillouin zone, and a third flat band with a quadratic band touching at $\mathbf{\Gamma}$.

A circularly-polarized pump field now couples to electrons via Peierls substitution $\mathbf{k} \to \mathbf{k} + \mathbf{A}(t)$, with units $\hbar = e = 1$. As there is no charge gap in the absence of interactions, we consider here the off-resonant high-frequency regime with Ω much larger than the electronic band width. At weak pump strengths, the effective Hamiltonian now follows as

$$\begin{aligned} h_{\mathrm{eff}}(\mathbf{k}) &= h_0(\mathbf{k}) - \frac{1}{\Omega}\left[h_1(\mathbf{k}),\ h_{-1}(\mathbf{k})\right] \\ &= -2 \begin{bmatrix} 0 & \tilde{t}\cos\left(\frac{\mathbf{a}_1\cdot\mathbf{k}}{2}\right) & \tilde{t}^\star\cos\left(\frac{\mathbf{a}_2\cdot\mathbf{k}}{2}\right) \\ \tilde{t}^\star\cos\left(\frac{\mathbf{a}_1\cdot\mathbf{k}}{2}\right) & 0 & \tilde{t}\cos\left(\frac{\mathbf{a}_3\cdot\mathbf{k}}{2}\right) \\ \tilde{t}\cos\left(\frac{\mathbf{a}_2\cdot\mathbf{k}}{2}\right) & \tilde{t}^\star\cos\left(\frac{\mathbf{a}_3\cdot\mathbf{k}}{2}\right) & 0 \end{bmatrix} - 2i\tilde{t}' \begin{bmatrix} 0 & \cos\left(\frac{(\mathbf{a}_2-\mathbf{a}_3)\cdot\mathbf{k}}{2}\right) & -\cos\left(\frac{(\mathbf{a}_1+\mathbf{a}_3)\cdot\mathbf{k}}{2}\right) \\ -\cos\left(\frac{(\mathbf{a}_2-\mathbf{a}_3)\cdot\mathbf{k}}{2}\right) & 0 & \cos\left(\frac{(\mathbf{a}_1+\mathbf{a}_2)\cdot\mathbf{k}}{2}\right) \\ \cos\left(\frac{(\mathbf{a}_1+\mathbf{a}_3)\cdot\mathbf{k}}{2}\right) & -\cos\left(\frac{(\mathbf{a}_1+\mathbf{a}_2)\cdot\mathbf{k}}{2}\right) & 0 \end{bmatrix} \end{aligned} \tag{2}$$

with lattice vectors $\mathbf{a}_1 = (1,\ 0)^T$, $\mathbf{a}_2 = (1/2,\ \sqrt{3}/2)^T$, and $h_m(\mathbf{k}) = \frac{\Omega}{2\pi}\int_0^{2\pi/\Omega} dt\ e^{im\Omega t} h(\mathbf{k}+\mathbf{A}(t))$. The effective Hamiltonian is now parameterized by time-reversal symmetry breaking nearest and next-nearest neighbor hoppings

$$\tilde{t} = t_{\mathrm{h}}\left(1 - \frac{A^2}{4}\right) - i\frac{\sqrt{3}t_{\mathrm{h}}^2 A^2}{4\Omega} \tag{3}$$

$$\tilde{t}' = \frac{\sqrt{3}t_{\mathrm{h}}^2 A^2}{4\Omega} \tag{4}$$

where A is the dimensionless field strength as defined in the main text. Time-reversal symmetry breaking entails that a gap opens up at the two Dirac points at $\mathbf{K}, \mathbf{K}'$, however the quadratic band touching at $\mathbf{\Gamma}$ persists to lowest order in A.

Supplementary Note 2. QUASI-DEGENERATE PERTURBATION THEORY FOR THE DRIVEN KAGOME HUBBARD MODEL

Consider now a strongly-interacting Mott-Hubbard insulator on the Kagome lattice, driven by circularly-polarized light. To simplify notation, we consider a generic driven Hubbard model

$$\hat{H}(\tau) = -\sum_{ij\sigma} t_{ij}(\tau)\ \hat{c}^\dagger_{i\sigma}\hat{c}_{j\sigma} + U\sum_i \hat{n}_{i\uparrow}\hat{n}_{i\downarrow} \tag{5}$$

where the external drive enters in the time-dependent hopping amplitudes, and we denote time by τ. The case of external drive via Peierls substitution corresponds to $t_{ij}(\tau) = t_{\mathrm{h}} e^{i\mathbf{A}(\tau)\cdot\mathbf{r}_{ij}}$ as discussed in the main text. In Floquet language, one can similarly write:

$$\hat{H} = -\sum_{\substack{ij\sigma \\ mm'}} t_{ij}^{(m-n)} \hat{c}_{i\sigma}^{\dagger} \hat{c}_{j\sigma} \otimes |m\rangle\langle m'| + U\sum_{i} \hat{n}_{i\uparrow}\hat{n}_{i\downarrow} \otimes \mathbf{1} - \sum_{m} m\Omega\ \mathbf{1} \otimes |m\rangle\langle m| \tag{6}$$

where $t_{ij}^{(m-n)} = \int_0^{2\pi/\Omega} d\tau\ e^{i(m-n)\tau} t_{ij}(\tau)$ are Floquet hopping amplitudes that obey $\left(t_{ji}^{(-m)}\right)^{\star} = t_{ij}^{(m)}$.

So far, this description is exact. As described in the main text, the Hamiltonian retains a local moment description of interacting spins as long as U is much larger than hopping, and the pump is off-resonant with charge excitations. A spin description for pumping below the charge gap then follows from *simultaneously* integrating out doubly-occupied many-body states and photon (Floquet) side bands, treating strong-coupling and Floquet energy scales on equal footing.

Before discussing the derivation in detail, it is instructive to compare our methodology to the Schrieffer-Wolff transformation for driven systems[4]. The methods are *a priori* equivalent for pumping off the charge resonance (the regime of interest in this work), which is to be expected, as the perturbation expansion should give the same results independent of choice of basis. Importantly however, as discussed in detail below, the lowest-order contribution in virtual hopping merely serves to "renormalize" the strength of nearest-neighbor spin exchange interactions[4,5], which *cannot change the transient many-body state* as long as total spin is conserved. Instead, as discussed in the main text, the subleading photon-assisted contributions – to fourth order in virtual hopping – are essential to manipulate the equilibrium state, break time-reversal symmetry, and ultimately stabilize a chiral spin liquid.

Second Order in Virtual Hopping

In Floquet language, simultaneously integrating out charge and Floquet degrees of freedom as discussed above amounts to applying standard quasi-degenerate perturbation theory to the time-independent Floquet Hamiltonian in frequency formulation. We constrain ourselves to the half-filled Mott insulator. To second order in virtual hopping processes, the perturbation theory entails a renormalization of nearest-neighbor Heisenberg exchange interactions

$$\begin{aligned} \hat{H}^{(\text{2nd order})} &= \sum_{ij\sigma\sigma'} \frac{t_{ij}^{(-m)} t_{ji}^{(m)}}{U + m\Omega}\ \hat{c}_{i\sigma}^{\dagger}\hat{c}_{j\sigma}\hat{c}_{j\sigma'}^{\dagger}\hat{c}_{i\sigma'} \\ &= 2\sum_{\langle ij\rangle\sigma\sigma'} \frac{\left|t_{ji}^{(m)}\right|^2}{U + m\Omega}\ \hat{c}_{i\sigma}^{\dagger}\hat{c}_{i\sigma'}\hat{c}_{j\sigma}\hat{c}_{j\sigma'}^{\dagger} \\ &= 4\sum_{\langle ij\rangle} \frac{\left|t_{ji}^{(m)}\right|^2}{U + m\Omega}\ \mathbf{S}_i \cdot \mathbf{S}_j \end{aligned} \tag{7}$$

where the last line follows from a canonical change to spin-1/2 operators and the local moment constraint. Choosing Floquet hopping amplitudes $t_{ij}^{(m)}$ that correspond to circularly-polarized light finally leads to the result quoted in the main text.

Third Order in Virtual Hopping

As a short digression, a more interesting scenario appears to third-order in virtual hopping. In equilibrium, it is well-known that the third-order expansion vanishes in the presence of time-reversal symmetry. Conversely, if time-reversal symmetry is broken via an external magnetic field, a scalar spin chirality term appears already to this order, and is proportional to the phase acquired by electrons hopping around a triangle. Naïvely, a similar scenario should then arise already to third order, in the presence of a circularly-polarized field, with electrons acquiring a phase when absorbing or emitting photons during virtual hopping. Here, we show that this is not the case.

To third order in virtual hopping, and without any assumptions on symmetries of the problem, the generic fermionic

starting point of the expansion reads

$$\hat{H}^{(3)} = \sum_{\substack{ijk\\ \sigma\sigma'\sigma''\\ m_1m_2}} \frac{1}{(U+m_1\Omega)(U+m_2\Omega)} \Big[\hat{c}^\dagger_{i\sigma}\hat{c}_{j\sigma}\hat{c}^\dagger_{j\sigma'}\hat{c}_{k\sigma'}\hat{c}^\dagger_{k\sigma''}\hat{c}_{i\sigma''}t^{(-m_2)}_{ij}t^{(m_2-m_1)}_{jk}t^{(m_1)}_{ki} + \\ + \hat{c}^\dagger_{j\sigma'}\hat{c}_{k\sigma'}\hat{c}^\dagger_{i\sigma}\hat{c}_{j\sigma}\hat{c}^\dagger_{k\sigma''}\hat{c}_{i\sigma''}t^{(-m_2)}_{jk}t^{(m_2-m_1)}_{ij}t^{(m_1)}_{ki}\Big] \tag{8}$$

where i, j, k sum over all sites. After appropriate expansion of all permutations, reordering of fermionic operators and recasting in terms of spin-1/2 operators, one arrives at a Heisenberg-chiral spin Hamiltonian

$$\hat{H}^{\text{3rd order}} = \sum_{\langle ij\rangle} J^{(\text{3rd order})}\ \mathbf{S}_i\cdot\mathbf{S}_j + \sum_{\substack{\triangle\\ ijk}} \chi^{(\text{3rd order})}\ \mathbf{S}_i\cdot(\mathbf{S}_j\times\mathbf{S}_k) \tag{9}$$

Here, the Heisenberg exchange coupling and scalar spin chirality coupling can be expressed as

$$\chi^{\text{3rd order}} = \sum_{m_1m_2} \frac{2}{(U+m_1\Omega)(U+m_2\Omega)} \mathrm{Im}\Big[t^{(m_2)}_{ij}t^{(m_1-m_2)}_{jk}t^{(-m_1)}_{ki} + t^{(m_1-m_2)}_{ij}t^{(m_2)}_{jk}t^{(-m_1)}_{ki} + t^{(m_2-m_1)}_{ij}t^{(-m_2)}_{jk}t^{(m_1)}_{ki} + \\ + t^{(-m_2)}_{ij}t^{(m_2-m_1)}_{jk}t^{(m_1)}_{ki} + t^{(m_2)}_{ij}t^{(-m_1)}_{jk}t^{(m_1-m_2)}_{ki} + t^{(-m_1)}_{ij}t^{(m_2)}_{jk}t^{(m_1-m_2)}_{ki} + t^{(m_2-m_1)}_{ij}t^{(m_1)}_{jk}t^{(-m_2)}_{ki} + \\ + t^{(m_1)}_{ij}t^{(m_2-m_1)}_{jk}t^{(-m_2)}_{ki} + t^{(m_1-m_2)}_{ij}t^{(-m_1)}_{jk}t^{(m_2)}_{ki} + t^{(-m_1)}_{ij}t^{(m_1-m_2)}_{jk}t^{(m_2)}_{ki} + t^{(-m_2)}_{ij}t^{(m_1)}_{jk}t^{(m_2-m_1)}_{ki} + \\ + t^{(m_1)}_{ij}t^{(-m_2)}_{jk}t^{(m_2-m_1)}_{ki}\Big] \tag{10}$$

$$J^{\text{3rd order}} = \sum_{m_1m_2} \frac{1}{(U+m_1\Omega)(U+m_2\Omega)} \mathrm{Re}\Big[-(t^{(m_2)}_{ij}t^{(m_1-m_2)}_{jk}t^{(-m_1)}_{ki}) - t^{(m_1-m_2)}_{ij}t^{(m_2)}_{jk}t^{(-m_1)}_{ki} + t^{(m_2-m_1)}_{ij}t^{(-m_2)}_{jk}t^{(m_1)}_{ki} + \\ + t^{(-m_2)}_{ij}t^{(m_2-m_1)}_{jk}t^{(m_1)}_{ki} + t^{(m_2)}_{ij}t^{(-m_1)}_{jk}t^{(m_1-m_2)}_{ki} - t^{(-m_1)}_{ij}t^{(m_2)}_{jk}t^{(m_1-m_2)}_{ki} - t^{(m_2-m_1)}_{ij}t^{(m_1)}_{jk}t^{(-m_2)}_{ki} - \\ - t^{(m_1)}_{ij}t^{(m_2-m_1)}_{jk}t^{(-m_2)}_{ki} + t^{(m_1-m_2)}_{ij}t^{(-m_1)}_{jk}t^{(m_2)}_{ki} + t^{(-m_1)}_{ij}t^{(m_1-m_2)}_{jk}t^{(m_2)}_{ki} - t^{(-m_2)}_{ij}t^{(m_1)}_{jk}t^{(m_2-m_1)}_{ki} + \\ + t^{(m_1)}_{ij}t^{(-m_2)}_{jk}t^{(m_2-m_1)}_{ki}\ \Big] \tag{11}$$

To proceed, consider a generic parameterization of the Floquet hoppings

$$t^{(m)}_{ij} = t_{ij}\mathcal{J}_m(A_{ij})e^{im\psi_{ij}} \tag{12}$$

Importantly, one then finds that the terms in the bracket in Supplementary Eq. (10) are real and the chiral contribution $\chi^{\text{3rd order}}$ *vanishes exactly even if time-reversal symmetry is broken* is by the external field. In other words, the photon-mediated phases acquired by electrons hopping around a triangle remarkably cancel exactly and to all orders in pump strength A. The third-order Heisenberg contribution $J^{\text{3rd order}}$ vanishes analogously, with the terms in the bracket in Supplementary Eq. (11) purely imaginary.

Instead, in analogy to the equilibrium case, solely a *static* magnetic field will induce a scalar spin chirality term to third order. As discussed in the main text however, in this case the Zeeman shift will generically dominate for an external magnetic field and preclude the formation of a chiral spin liquid.

For completeness, we could now consider the joint effect of a *static* magnetic field and time-dependent circularly-polarized pump. Here, the nearest-neighbor Floquet hopping amplitudes

$$t^{(m)}_{\langle ij\rangle} = e^{i\phi_{\text{mag}}}\ t_{\text{h}}\mathcal{J}_m(A_{ij})e^{im\psi_{ij}} \tag{13}$$

would acquire an additional phase ϕ_{mag} due to the static magnetic flux through the triangle. Unlike the equilibrium problem, the third-order contribution to the effective Hamiltonian in this case entails not only a scalar spin chirality term but further corrections to nearest-neighbor Heisenberg exchange

$$J^{\text{3rd order}} = \sum_{m_1m_2} \frac{4t_{\text{h}}^3\mathcal{J}_{m_1}(A)\mathcal{J}_{m_2-m_1}(A)\mathcal{J}_{-m_2}(A)}{(U+m_1\Omega)(U+m_2\Omega)} \sin\left[\tfrac{2\pi(2m_1-m_2)}{3}\right]\ \sin(3\phi_{\text{mag}}) \tag{14}$$

$$\chi^{\text{3rd order}} = \sum_{m_1m_2} \frac{24t_{\text{h}}^3\mathcal{J}_{m_1}(A)\mathcal{J}_{m_2-m_1}(A)\mathcal{J}_{-m_2}(A)}{(U+m_1\Omega)(U+m_2\Omega)} \cos\left[\tfrac{2\pi(2m_1-m_2)}{3}\right]\ \sin(3\phi_{\text{mag}}) \tag{15}$$

The well-known equilibrium result[1–3] for solely a static magnetic field can be readily recovered setting A to zero, which yields $J^{\text{3rd order}} = 0$ and $\chi^{\text{3rd order}} = 24t_{\text{h}}^3/U^2 \sin(3\phi_{\text{mag}})$.

Fourth Order in Virtual Hopping

Constituting the central result of the main text, dynamical time-reversal symmetry breaking due to circularly-polarized light first enters the effective spin description to fourth order in photon-assisted virtual hopping processes. The presence and relevance of this effect in determining the transient steady state is remarkable – whereas fourth-order contributions to spin interactions will commonly be negligible in equilibrium Mott insulators, the out-of-equilibrium problem provides a powerful knob to tune the spin system into a regime where such contributions emerge, in fact, as the determining mechanism to stabilize the transient phase.

The quasi-degenerate perturbation theory again proceeds as usual, however entails a large number of combinatoric contributions due to the various permutations of hopping virtually through different Floquet side bands. We therefore developed a computer algebra program to perform the perturbation expansion and recasting into spin language. Here, we solely quote the fermionic starting point of the fourth-order expansion

$$\begin{aligned}
\hat{H}^{(4)} = &- \sum_{\substack{ijkl \\ \sigma\sigma'\sigma''\sigma''' \\ m_1 m_2 m_3}} \Bigg[\hat{c}^\dagger_{i\sigma}\hat{c}_{j\sigma}\hat{c}^\dagger_{j\sigma'}\hat{c}_{k\sigma'}\hat{c}^\dagger_{k\sigma''}\hat{c}_{l\sigma''}\hat{c}^\dagger_{l\sigma'''}\hat{c}_{i\sigma'''} \frac{(1-\delta_{ik}\delta_{m_2})\; t^{(-m_3)}_{ij} t^{(m_3-m_2)}_{jk} t^{(m_2-m_1)}_{kl} t^{(m_1)}_{li}}{(U+m_1\Omega)(U(1-\delta_{ik})+m_2\Omega)(U+m_3\Omega)} + \\
&+ \hat{c}^\dagger_{j\sigma'}\hat{c}_{k\sigma'}\hat{c}^\dagger_{i\sigma}\hat{c}_{j\sigma}\hat{c}^\dagger_{k\sigma''}\hat{c}_{l\sigma''}\hat{c}^\dagger_{l\sigma'''}\hat{c}_{i\sigma'''} \frac{(1-\delta_{ik}\delta_{m_2})\; t^{(-m_3)}_{jk} t^{(m_3-m_2)}_{ij} t^{(m_2-m_1)}_{kl} t^{(m_1)}_{li}}{(U+m_1\Omega)(U(1-\delta_{ik})+m_2\Omega)(U+m_3\Omega)} + \\
&+ \hat{c}^\dagger_{j\sigma'}\hat{c}_{k\sigma'}\hat{c}^\dagger_{k\sigma''}\hat{c}_{l\sigma''}\hat{c}^\dagger_{i\sigma}\hat{c}_{j\sigma}\hat{c}^\dagger_{l\sigma'''}\hat{c}_{i\sigma'''} \frac{(1-\delta_{jl}\delta_{m_2})\; t^{(-m_3)}_{jk} t^{(m_3-m_2)}_{kl} t^{(m_2-m_1)}_{ij} t^{(m_1)}_{li}}{(U+m_1\Omega)(U(1-\delta_{jl})+m_2\Omega)(U+m_3\Omega)} + \\
&+ \hat{c}^\dagger_{k\sigma''}\hat{c}_{l\sigma''}\hat{c}^\dagger_{j\sigma'}\hat{c}_{k\sigma'}\hat{c}^\dagger_{i\sigma}\hat{c}_{j\sigma}\hat{c}^\dagger_{l\sigma'''}\hat{c}_{i\sigma'''} \frac{(1-\delta_{jl}\delta_{m_2})\; t^{(-m_3)}_{kl} t^{(m_3-m_2)}_{jk} t^{(m_2-m_1)}_{ij} t^{(m_1)}_{li}}{(U+m_1\Omega)(U(1-\delta_{jl})+m_2\Omega)(U+m_3\Omega)} \Bigg] + \\
&- \sum_{\substack{ijkl \\ \sigma\sigma'\sigma''\sigma''' \\ m_1 m_2 m_3}} \frac{1}{(U+m_1\Omega)(2U+m_2\Omega)(U+m_3\Omega)} \Big[\hat{c}^\dagger_{i\sigma}\hat{c}_{l\sigma}\hat{c}^\dagger_{j\sigma'}\hat{c}_{k\sigma'}\hat{c}^\dagger_{k\sigma''}\hat{c}_{j\sigma''}\hat{c}^\dagger_{l\sigma'''}\hat{c}_{i\sigma'''} t^{(-m_3)}_{il} t^{(m_3-m_2)}_{jk} t^{(m_2-m_1)}_{kj} t^{(m_1)}_{li} + \\
&+ \hat{c}^\dagger_{j\sigma}\hat{c}_{l\sigma}\hat{c}^\dagger_{i\sigma'}\hat{c}_{k\sigma'}\hat{c}^\dagger_{k\sigma''}\hat{c}_{j\sigma''}\hat{c}^\dagger_{l\sigma'''}\hat{c}_{i\sigma'''} t^{(-m_3)}_{jl} t^{(m_3-m_2)}_{ik} t^{(m_2-m_1)}_{kj} t^{(m_1)}_{li} \\
&+ \hat{c}^\dagger_{j\sigma}\hat{c}_{k\sigma}\hat{c}^\dagger_{i\sigma'}\hat{c}_{l\sigma'}\hat{c}^\dagger_{k\sigma''}\hat{c}_{j\sigma''}\hat{c}^\dagger_{l\sigma'''}\hat{c}_{i\sigma'''} t^{(-m_3)}_{jk} t^{(m_3-m_2)}_{il} t^{(m_2-m_1)}_{kj} t^{(m_1)}_{li} \\
&+ \hat{c}^\dagger_{i\sigma}\hat{c}_{k\sigma}\hat{c}^\dagger_{j\sigma'}\hat{c}_{l\sigma'}\hat{c}^\dagger_{k\sigma''}\hat{c}_{j\sigma''}\hat{c}^\dagger_{l\sigma'''}\hat{c}_{i\sigma'''} t^{(-m_3)}_{ik} t^{(m_3-m_2)}_{jl} t^{(m_2-m_1)}_{kj} t^{(m_1)}_{li} \Big] \\
&+ \frac{1}{2} \sum_{\substack{ijkl \\ \sigma\sigma'\sigma''\sigma''' \\ m_1 m_2}} \hat{c}^\dagger_{k\sigma}\hat{c}_{l\sigma}\hat{c}^\dagger_{l\sigma'}\hat{c}_{k\sigma'}\hat{c}^\dagger_{i\sigma''}\hat{c}_{j\sigma''}\hat{c}^\dagger_{j\sigma'''}\hat{c}_{i\sigma'''}\; t^{(-m_2)}_{kl} t^{(m_2)}_{lk} t^{(-m_1)}_{ij} t^{(m_1)}_{ji} \Bigg[\frac{1}{(U+m_1\Omega)(U+m_2\Omega)^2} + \\
&+ \frac{1}{(U+m_1\Omega)^2(U+m_2\Omega)} \Bigg]
\end{aligned} \tag{16}$$

where we imply within the terms of the first sum that $\frac{(1-\delta_{ik}\delta_{m_2})}{U(1-\delta_{ik})+m_2\Omega} = 0$ if $i = k$ and $m_2 = 0$. The resulting spin Hamiltonian for circularly-polarized pumping can now be derived from tedious but straightforward algebraic operations, and is presented in the main text.

We note that similar perturbative expansions must be used to recast electronic experimental observables in terms of the effective low-energy spin degrees of freedom. A detailed discussion of optical and Raman probes (in particular, the A_{2g} channel and its analogue for the Kagome lattice) to fourth order in virtual hopping processes will be discussed in an upcoming publication.

Supplementary Note 3. PUMP-ENVELOPE DEPENDENCE AND MANY-BODY DYNAMICS OF THE DRIVEN KAGOME-HUBBARD MODEL

The main text investigates the many-body dynamics of a Kagome-Hubbard model pumped with circularly-polarized light, and compares the driven steady state at the pump plateau to the effective Floquet chiral spin model that is the main focus of this work. To extract scalar spin chirality expectation values from the driven steady state at the pump

plateau, we consider pump fields of the form

$$\mathbf{A}(t) = A(t) \begin{bmatrix} \cos(\Omega t) \\ \sin(\Omega t) \end{bmatrix} \tag{17}$$

with a smooth sinusoidal pump envelope

$$A(t) = \begin{cases} 0, & t \leq 0 \\ A \sin^2\left(\frac{\pi}{2} \frac{t}{t_{\text{plateau}}}\right), & 0 < t < t_{\text{plateau}} \\ A, & t \geq t_{\text{plateau}} \end{cases} \tag{18}$$

with $t_{\text{plateau}} = 500 t_{\text{h}}^{-1}$. Supplementary Fig. **1** depicts the period-averaged scalar spin chirality expectation values $\langle \chi_{ijk} \rangle$ for three distinct triangles of the Kagome unit cell, as well as a the raw dynamical data (including micro-motion within the pump periods), close to the charge resonance for a 12-site cluster. Fig. 4(c) of the main text now follows straight-forwardly via extracting the average expectation values $\langle \chi_{ijk} \rangle$ at the pump plateau.

To analyze the influence of the pump envelope on controlled preparation of the steady state, we calculate the scalar spin chirality expectation values as well as double occupancies and period-averaged energy, as a function of t_{plateau}, i.e. as a function of ramp-up speed. Supplementary Fig. **2** depicts the envelope dependence for scalar spin chirality expectation values, showing excellent control of the transient steady state for the pump envelope widths used in the main text. To consider residual effects of heating and energy absorption, Supplementary Fig. **3** depicts transient double occupancies and period-averaged energy on the pump plateau, as a function of pump envelope width, for intermediate and strong pumping. Analogously, the steady state is remarkably robust, with heating strongly suppressed. To quantify the latter, residual energy absorption rates can be extracted from the gradient of the period-averaged energy, and are depicted in Fig. 5(e) of the main text.

$$A(t) = \begin{cases} 0, & t \leq 0 \\ A \sin^2\left(\frac{\pi}{2} \frac{t}{t_{\text{plateau}}}\right), & 0 < t < t_{\text{plateau}} \\ A, & t \geq t_{\text{plateau}} \end{cases} \tag{19}$$

The parameters chosen in the main text correspond to $t_{\text{plateau}} = 500 t_{\text{h}}^{-1}$.

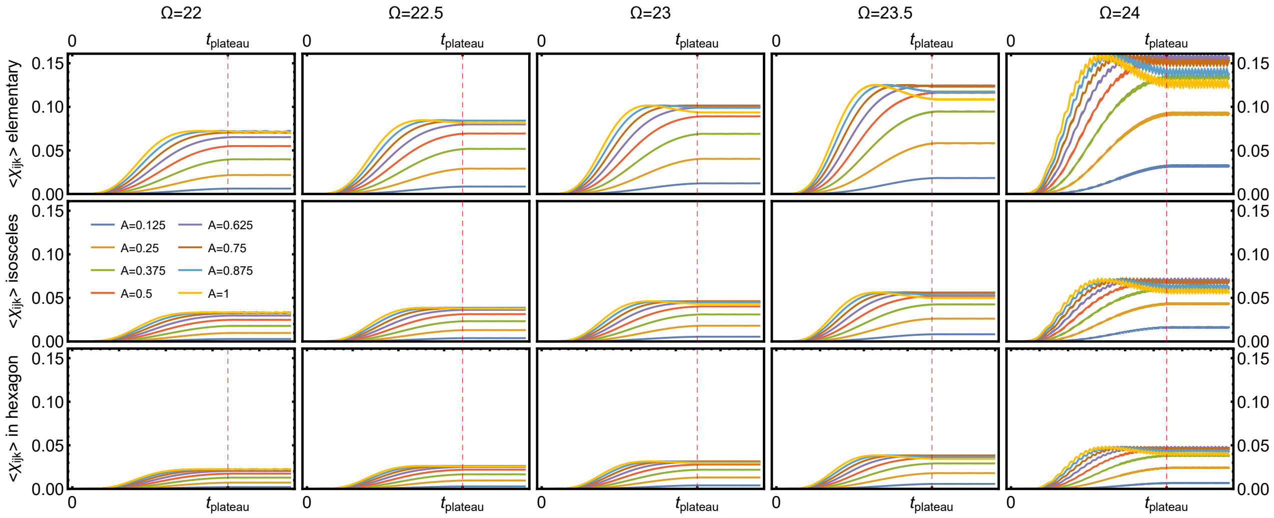

(a) Period-averaged scalar spin chirality expectation values.

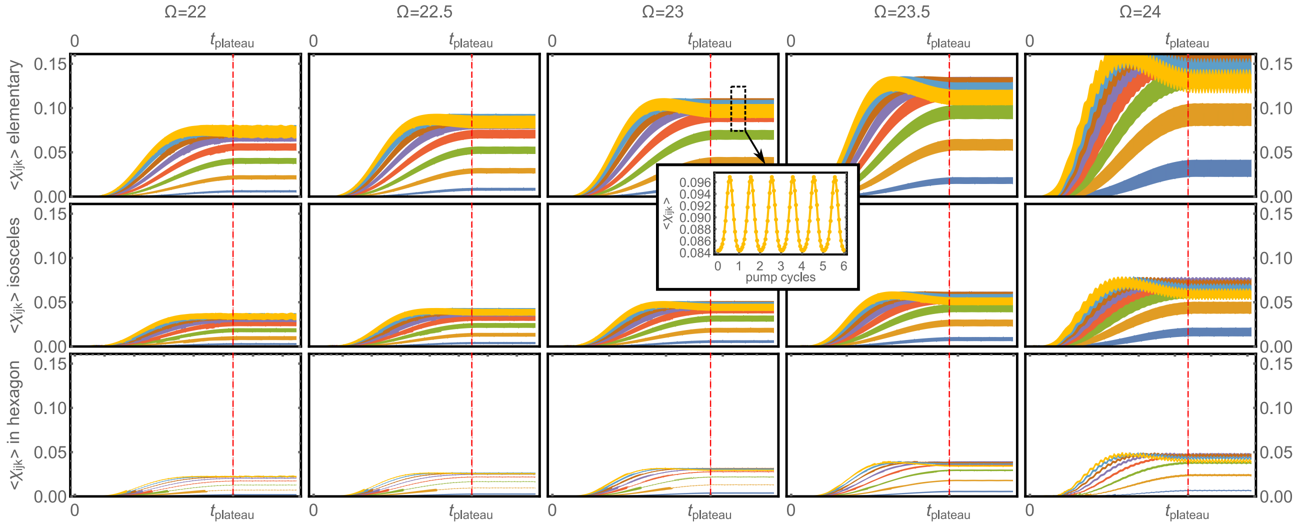

(b) Raw dynamical data.

Supplementary Figure 1. **Time evolution of scalar spin chirality expectation values.** Panels depict time-dependent expectation values $\langle \mathbf{S}_i \cdot (\mathbf{S}_j \times \mathbf{S}_k)\,(t)\rangle$ for circularly-polarized pumping close to the one-photon charge resonance, as a function of pump strength and frequency. (a) depicts a triplet of rows corresponding to measuring the period-averaged scalar spin chirality on three triangles in the unit cell: elementary triangles of the Kagome lattice, isosceles triangles inside the hexagon, and equilateral triangles inside the hexagon, as depicted graphically in Fig. 4 (b) of the main text. (b) depicts the corresponding raw dynamical data. The broadening of lines stems from micro-motion within individual pump periods, shown in detail in the inset. Floquet expectation values are recovered by averaged out the micro-motion, as shown in (a).

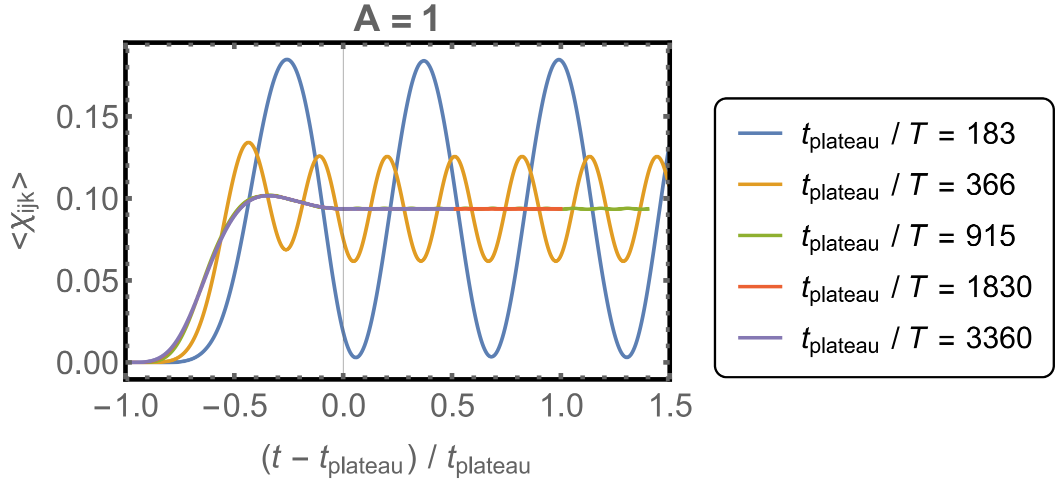

Supplementary Figure 2. **Pump envelope dependence of scalar spin chirality expectation values**. The time evolution of period-averaged scalar spin chirality expectation values is shown for elementary triangles of the Kagome lattice, for $\Omega = 23 t_{\mathrm{h}}$. Here, t_{plateau}/T quantifies the number of pump cycles under the ramp-up, before reaching the pump plateau at time $t = t_{\mathrm{plateau}}$ (time axis is normalized accordingly). Pump envelopes used for Fig. 4 and Fig. 5 of the main text are depicted in green ($t_{\mathrm{plateau}}/T = 915$).

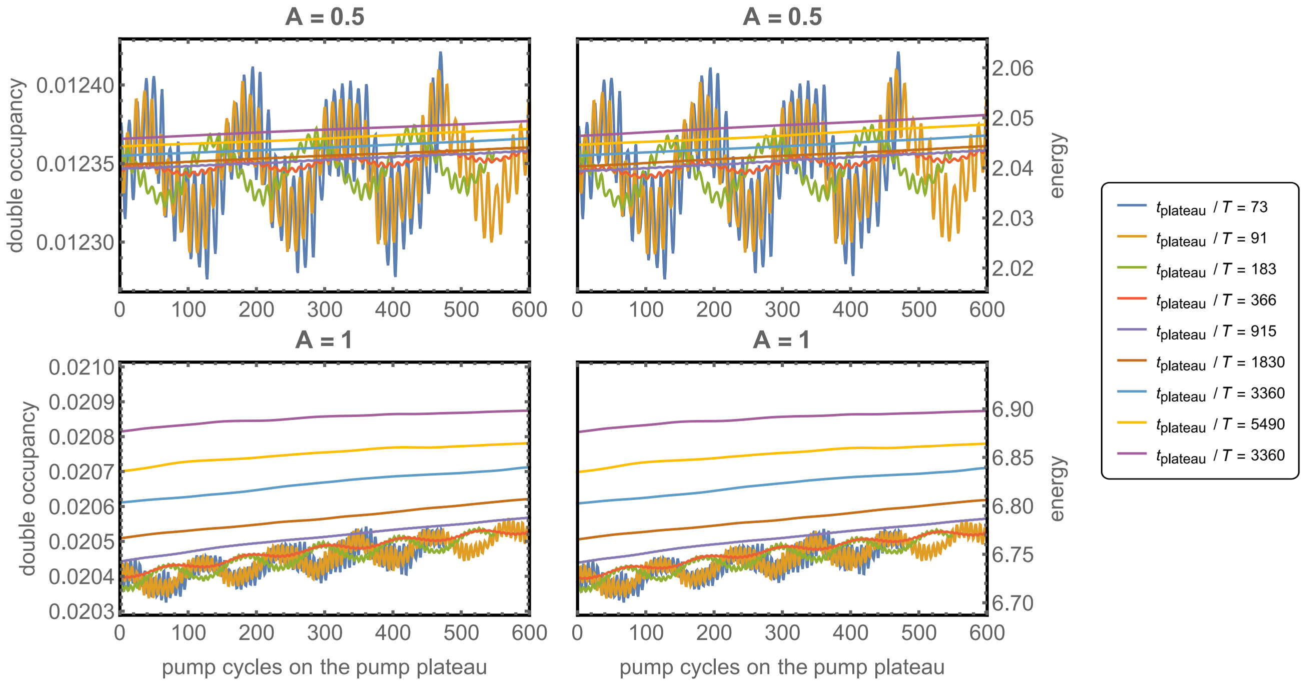

Supplementary Figure 3. **Pump envelope dependence of period-averaged double occupancies and energy.** Tow (bottom) rows correspond to intermedite (strong) pumping, for $\Omega = 23 t_{\mathrm{h}}$, on the pump plateau (pump cycle $0 \equiv t_{\mathrm{plateau}}$). Energy depicted in units of hopping strength. The time derivative of double occupancies and energy yields effective heating rates, demonstrating negligible heating both during the pump ramp-up and on the pump plateau, for pumping below the one-photon charge resonance.

SUPPLEMENTARY REFERENCES

[1] A. H. MacDonald, S. M. Girvin, and D. Yoshioka, Phys. Rev. B **37**, 9753 (1988).
[2] D. Sen, and R. Chitra, Phys. Rev. B **51**, 1922 (1995).
[3] O. I. Motrunich, Phys. Rev. B **73**, 155155 (2006).
[4] M. Bukov, M. Kolodrubetz, and A. Polkovnikov, Phys. Rev. Lett. **116**, 125301 (2016).
[5] J. H. Mentink, K. Balzer, and M. Eckstein, Nature Comm. **6**, 6708 (2015).
